# Don’t Get It Twisted: A Case of Pediatric Sigmoid Volvulus Presenting as Lower Back Pain

**DOI:** 10.7759/cureus.109498

**Published:** 2026-05-23

**Authors:** Zachary N Kossiver, Rachel E Bridwell, Jared Carter, Hannah Kobit

**Affiliations:** 1 Medicine, University of Tampa, Tampa, USA; 2 Emergency Medicine, Atrium Health, Charlotte, USA; 3 Emergency Medicine, Womack Army Medical Center, Fayetteville, USA

**Keywords:** emergency, idiopathic pediatric sigmoid volvulus, large bowel obstruction, pediatric rare diseases, rare case, redundant sigmoid colon, sigmoid volvulus

## Abstract

Sigmoid volvulus (SV) is a closed‑loop large bowel obstruction that is exceedingly uncommon in children and adolescents.Early diagnosis is essential, as progressive torsion of a redundant sigmoid colon around its mesentery leads to venous congestion, arterial compromise, and eventual bowel necrosis and perforation if untreated.Pediatric SV presents with crampy abdominal pain, distension, vomiting, and obstipation; however, low back pain has not previously been described as a primary presenting symptom. This report describes the case of a 17‑year‑old boy who presented with a chief complaint of severe bilateral lower back and moderate bilateral hip and flank pain, as well as a previous bout of associated mild suprapubic discomfort that subsided prior to arrival. He was subsequently diagnosed with SV on computed tomography and was successfully managed with emergent endoscopic detorsion followed by elective robotic sigmoid resection.

## Introduction

Sigmoid volvulus (SV) occurs when a redundant sigmoid colon twists around its elongated mesenteric pedicle, creating a closed‑loop obstruction, which can rapidly progress to ischemia, gangrene, and perforation [1]. While SV is a more commonly encountered surgical emergency in older adults, it is rare in the pediatric population and lacks standardized diagnostic and management guidelines [2].

Reported pediatric cases, although rare, typically present with marked colicky abdominal pain, progressive distension, constipation, nausea, and vomiting, sometimes accompanied by diarrhea or fecal incontinence; however, musculoskeletal and spinal complaints such as low back pain have not been emphasized [2,3]. This atypical presentation may contribute to delays in recognition when children present with atypical symptom complexes that initially suggest alternative pathology rather than intra‑abdominal catastrophe [3].

This report describes the case of an otherwise healthy pediatric patient whose initial presentation consisted of bilateral lumbosacral, posterior hip, and lower flank pain, ultimately leading to the diagnosis of SV requiring surgical intervention. This report aims to heighten the awareness of emergency clinicians that pediatric SV can masquerade as predominantly low back pain and flank pain, underscoring the need to include intra‑abdominal catastrophe in the differential diagnosis when musculoskeletal explanations do not fully account for the severity or evolution of symptoms and physical exam findings.

## Case presentation

A previously healthy 17‑year‑old male patient presented to the emergency department (ED) at 17:30 with abrupt‑onset, severe, crampy pain localized to the bilateral sacroiliac regions and radiating to the bilateral trochanters and inguinal areas. Over the preceding two days, he had experienced progressively worsening bilateral posterior lower flank and lumbar discomfort, accompanied by loose stools and two episodes of bowel incontinence. Approximately 24 hours before presentation, he reported a single, brief episode of intermittent colicky suprapubic pain lasting less than 10 minutes, which resolved spontaneously. On the morning of presentation, he completed an approximately six‑hour commercial flight, and within one to two hours of disembarkation, his lumbosacral and bilateral lower flank pain acutely escalated to constant, 10‑out‑of‑10 intensity, refractory to over‑the‑counter ibuprofen taken at home, prompting ED evaluation. He denied nausea, vomiting, hematochezia, melena, dysuria, frequency, urgency, and constitutional symptoms. There was no history of similar episodes, previous abdominal surgery, trauma, renal calculi, chronic constipation, antidiarrheal or laxative use, or known family history of Hirschsprung disease, bowel obstruction, or volvulus.

On arrival, vital signs revealed a blood pressure of 148/86 mm Hg, heart rate of 112 beats per minute, respiratory rate of 17 breaths per minute, temperature of 99.2°F, and oxygen saturation 98% on room air. On examination, the abdomen was non-tender on palpation but tense and distended with marked tympany to percussion in all quadrants, most pronounced around the umbilicus, with significantly diminished bowel sounds. The patient exhibited severe bilateral low back pain, reproducible with palpation over the sacroiliac joints, without overlying erythema, ecchymosis, deformity, or step-offs. Neurologic examination of the lower extremities was normal, demonstrating intact strength, preserved sensation across L4-S1 dermatomes, normal motor function, symmetric patellar and Achilles reflexes, and negative straight-leg raise testing. However, the physical examination was partially limited secondary to the patient’s significant discomfort and persistent movement, despite administration of 8 mg of intravenous morphine prior to assessment. Digital rectal examination was deferred due to patient discomfort; no visible blood or prolapse was noted on visual inspection. A digital rectal exam was requested once again following analgesia; however, the patient again declined with concerns for discomfort. 

Laboratory testing demonstrated a normal complete blood count, serum electrolytes, renal function, liver transaminases, and serum lactate, with the exception of a mildly elevated alkaline phosphatase of 145 U/L (reference range 55-140 U/L), given the patient's age (Tables 1, 2).

**Table 1:** Complete blood count (CBC) H: high; L: low

Laboratory study	Patient value	Reference range
White blood cell count (WBC)	8.7	4.0 - 10.5 ×10^3/uL
Red blood cell count (RBC)	5.56	4.63 - 6.08 ×10^6/uL
Hemoglobin (Hgb)	16.0	13.7 - 17.5 g/dL
Hematocrit (Hct)	45.4	40.1 - 51.0 %
Mean corpuscular volume (MCV)	81.7	79.0 - 92.2 fL
Mean corpuscular hemoglobin (MCH)	28.8	25.7 - 32.2 pg
Mean corpuscular hemoglobin concentration (MCHC)	35.2	32.3 - 36.5 g/dL
Red cell distribution width (RDW)	11.7	11.6 - 14.4 %
Platelet count (Plt)	273	150 - 400 ×10^3/uL
Mean platelet volume (MPV)	9.1 L (within normal limits for patient age)	9.4 - 12.4 fL
Immature Gran %	0.3	0.0 - 0.4 %
Neutrophils %	62.9	34.0 - 67.9 %
Lymphocytes %	26.4	21.8 - 53.1 %
Monocytes %	6.7	5.3 - 12.2 %
Eosinophils %	3.2	0.8 - 7.0 %
Basophils %	0.5	0.2 - 1.2 %
Nucleated RBC %	0.0	0.0 - 2.0 %
Immature Gran #	0.03	0.00 - 0.03 ×10^3/uL
Neutrophils #	5.49 H (mildly elevated)	1.78 - 5.38 ×10^3/uL
Lymphocytes #	2.30	1.32 - 3.57 ×10^3/uL
Monocytes #	0.58	0.30 - 0.82 ×10^3/uL
Eosinophils #	0.28	0.04 - 0.54 ×10^3/uL
Basophils #	0.04	0.01-0.08 x10^3/uL
Nucleated RBCs #	0.00	0.00-0.18 x10^3/uL

**Table 2:** Complete metabolic panel *Chronic Kidney Disease Epidemiology (CKD-EPI) Collaboration, 2021 H: high; L: low; BUN: blood urea nitrogen; eGFR: estimated glomerular filtration

Laboratory study	Patient value	Reference range
Sodium	140	136 - 145 mmol/L
Potassium	3.6	3.6 - 5.2 mmol/L
Chloride	108	100 - 108 mmol/L
Carbon dioxide	27.0	21.0 - 32.0 mmol/L
Anion gap	5	5 - 15 mmol/L
BUN	12	7 - 18 mg/dL
Creatinine	1.20	0.55 - 1.30 mg/dL
eGFR rate*	87.7 L (within normal limits for patient age)	>90.0
BUN/Creatinine Ratio	10.0	5.0 - 26.0
Glucose (non-fasting)	111 H	70 - 110 mg/dL
Calculated Osmolality	279.4 L	280 - 300 mosm/kg
Lactic Acid	1.8	0.4 - 2.0 mmol/L
Calcium	9.7	8.8 - 10.5 mg/dL
Total Bilirubin	0.50	0.0 - 1.00 mg/dL
Aspartate aminotransferase	23	15 - 37 Units/L
Alanine aminotransferase	27 L (within normal limits for patient age)	30 - 65 Units/L
Alkaline phosphatase	145 H (within normal limits for patient age)	50 - 136 Units/L
Total protein	7.7	6.4 - 8.2 g/dL
Albumin	4.4	3.4 - 5.0 g/dL
Globulin	3.3	1.8 - 4.2 g/dL
Albumin/Globulin ratio	1.3	-

Given the patient’s atypical presentation, marked abdominal distension with tympany, limited physical examination, and a broad differential diagnosis, including but not limited to intra-abdominal and lower lumbar musculoskeletal pathology, early computed tomography (CT) imaging was pursued to further characterize the underlying disease process and exclude alternative intra-abdominal and retroperitoneal etiologies. CT scan of the abdomen and pelvis with IV contrast showed a massively dilated sigmoid loop with an inverted “coffee bean” configuration arising from the pelvis and extending toward the upper abdomen, consistent with SV without overt colonic perforation, ischemia, or pneumoperitoneum (Figure 1).

**Figure 1:**
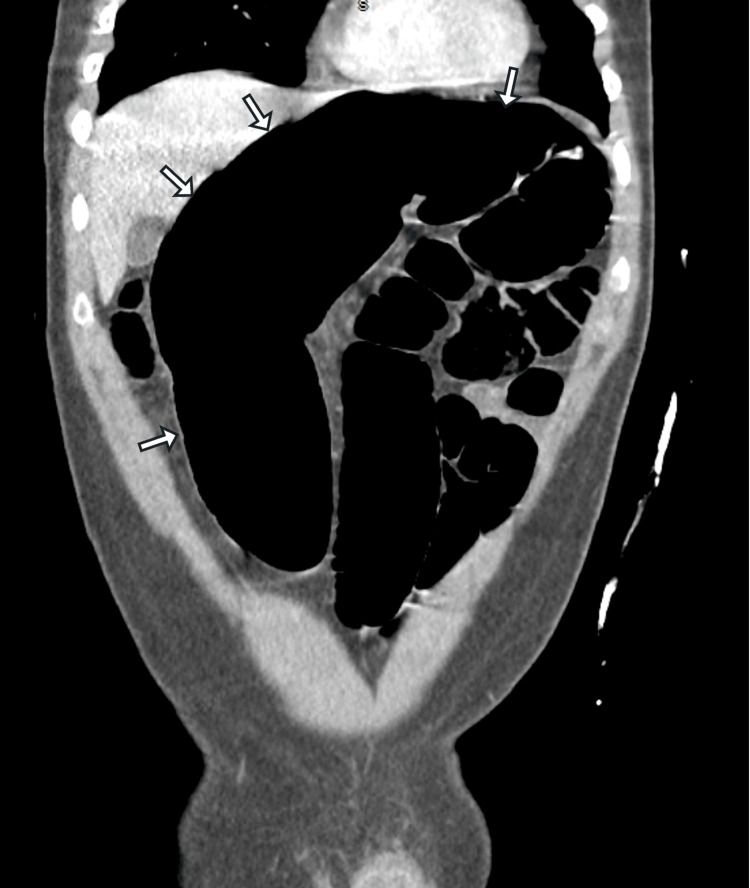
Computed tomography (CT) scan of the abdomen and pelvis with IV contrast A massively dilated sigmoid loop with an inverted “coffee bean” configuration (white arrows) is seen arising from the pelvis and extending toward the upper abdomen, consistent with sigmoid volvulus without overt colonic perforation, ischemia, or pneumoperitoneum

Based on the exam and imaging, both gastroenterology and general surgery were urgently consulted. In the absence of peritonitis and ischemia, the gastroenterology team performed an immediate flexible sigmoidoscopic decompression and detorsion under sedation, with periprocedural 1 g metronidazole and 1 g cefazolin. Endoscopy showed a distended, twisted sigmoid colon with viable mucosa, with successful detorsion.

On hospital day two, the patient underwent elective robotic‑assisted sigmoid colectomy with primary colorectal anastomosis after further resuscitation and stabilization. Intraoperatively, the sigmoid colon was found to be elongated and redundant with a narrow mesenteric attachment but without gross ischemia or perforation. Pathologic examination of the 32 cm of resected sigmoid colon confirmed viable colonic tissue with submucosal congestion and edema, consistent with recent volvulus, but no chronic inflammatory or neoplastic process (Figure 2).

**Figure 2:**
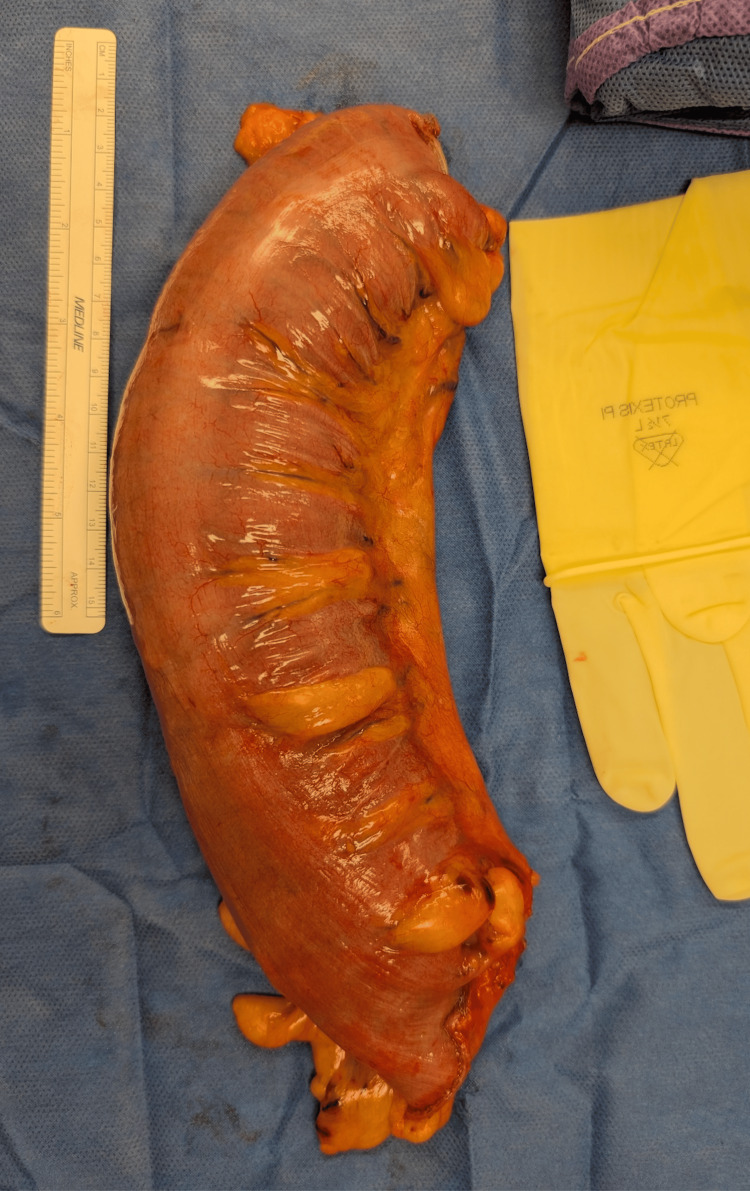
Gross specimen of resected sigmoid colon following surgery for sigmoid volvulus.

The postoperative course was uncomplicated, and the patient was discharged home on hospital day four after dietary advancement. At routine outpatient follow‑up six weeks later, he remained asymptomatic with normal bowel habits and without recurrence.

## Discussion

SV accounts for a substantial proportion of large bowel obstructions in adults [1,4]; however, it remains rare in children and adolescents. Sparse case series and systematic reviews describe its unique presentation in the pediatric demographic as a cause of mechanical ileus [1,4,5]. Since the 1940s, only about 100 pediatric cases have been documented, many originating from anatomical regions with higher baseline rates of colonic redundancy and volvulus, underscoring the dearth of data and, by extension, pediatric‑specific guidelines [1,6]. Predisposing anatomic factors include a long, redundant sigmoid colon with a narrow mesenteric base, elongated mesentery, and conditions associated with colonic dysmotility, such as intestinal malrotation, omphalomesenteric abnormalities, chronic constipation, Hirschsprung disease, and pseudo‑obstruction, although many adolescents, including the present patient, lack these risk factors [3,7].

Torsion of the sigmoid colon around its mesenteric axis produces a closed‑loop obstruction in which venous return is compromised before arterial inflow, leading to progressive venous congestion, mural edema, and eventual arterial ischemia, necrosis, and perforation [1,5]. The resulting proximal colonic distension increases intraluminal pressure and bowel wall tension, which, if unrelieved, may precipitate bacterial translocation, sepsis, and hemodynamic collapse [1,4,5]. Prompt recognition and decompression are therefore essential to prevent irreversible ischemic damage and reduce mortality, which increases sharply when perforation or generalized peritonitis occurs [8-10].

In pediatric cohorts, the predominant presenting symptoms of SV are recurrent or acute colicky abdominal pain, progressive abdominal distension, vomiting, constipation or obstipation, and occasionally diarrhea or rectal bleeding [2,3]. A systematic review of pediatric SV cases reported abdominal pain in approximately 85% of children, vomiting in 59%, constipation in 56%, and diarrhea in 10%, highlighting abdominal symptoms over referred symptoms as the presenting complaint [7]. Back pain has rarely been reported, and, based on the current literature, isolated bilateral lumbosacral pain as the chief complaint in the presence of only subtle recent transient suprapubic symptoms has not been previously highlighted in the pediatric SV literature [8,9]. 

Timely diagnosis of SV in children hinges upon integrating careful clinical assessment with appropriate imaging, recognizing that classic radiographic signs may be absent in a substantial minority of cases. Plain abdominal radiography can be obtained rapidly and, when diagnostic, reveals a markedly dilated, inverted‑U sigmoid loop pointing toward the upper abdomen, often described as the “coffee bean,” “kidney bean,” or “bent inner tube” sign; however, pediatric series indicate that plain films are diagnostic in only about 60% of cases and may miss smaller or atypically oriented volvuli, often necessitating CT imaging [6,8,9]. Given the patient’s severe pain, abdominal distension, and atypical presentation, there was great clinical concern for an acute intra-abdominal emergency, prompting immediate escalation to stat CT imaging in coordination with ED staff and radiology, thereby bypassing plain radiography to avoid delays in definitive diagnosis.

Contrast-enhanced CT of the abdomen and pelvis has become the imaging modality of choice when plain films are inconclusive or when there is suspicion for ischemia, given its ability to delineate both the degree of colonic distension and secondary signs such as mesenteric “whirl,” transition points, pneumatosis, or free air [2,8-10]. CT studies in the mixed‑aged populations report sensitivities of 80-90% for characteristic features of SV, including a distended sigmoid loop with a coffee-bean configuration, convergence of twisted mesenteric vessels forming a whirl sign, and abrupt cutoff of contrast or gas at the torsion site [6,8,9]. In children, cross‑sectional imaging also helps exclude alternative diagnoses such as malrotation with midgut volvulus, intussusception, or neoplastic obstruction, and it informs the urgency and type of intervention by identifying radiologic markers of ischemia or perforation [10]. Barium- or water‑soluble contrast enema retains a role in both diagnosis and, in some settings, therapeutic decompression, producing a tapering configuration at the transition point; however, its utilization has declined with the broader availability of CT and concerns about contrast extravasation in unrecognized perforation [8-10].

The mainstay of ED management includes resuscitation with crystalloids, early administration of parenteral opioids and antiemetics for analgesia and symptom relief, and broad-spectrum intravenous antibiotics (e.g., ceftriaxone and metronidazole or piperacillin-tazobactam) if there is concern for ischemia, a systemic inflammatory response, or impending perforation, to ensure appropriate microbe coverage [9,10].

Further management of SV in the absence of perforation or peritonitis generally follows a staged approach that prioritizes rapid decompression while planning for definitive resection during the same admission to minimize recurrence risk [10-12]. In both adult and pediatric populations, initial non‑operative detorsion using flexible sigmoidoscopy or contrast enema can be successful in 60-95% of cases, rapidly relieving obstruction and allowing assessment of mucosal viability, but recurrence rates after decompression alone are substantial, ranging from 30% to more than 60% without subsequent resection; exploratory laparotomy is reserved for clinically less stable patients [10,11]. Contemporary guidelines from the World Society of Emergency Surgery and pediatric case series therefore recommend elective sigmoid resection with primary anastomosis or Hartmann procedure after successful endoscopic decompression, particularly in otherwise healthy patients, to definitively address the underlying anatomic predisposition [2,8,10-14].

Emergency surgery is mandatory for patients with suspected or confirmed perforation, diffuse peritonitis, hemodynamic instability, or failed endoscopic detorsion, and it carries higher morbidity and mortality than semi‑elective resection performed after stabilization [15,16]. Clinical features such as suspected ischemia, marked colonic distension, delayed presentation, or unsuccessful endoscopic detorsion are associated with higher failure rates and should prompt consideration of early operative management [10,11,13]. Adult series have reported mortality rates approaching 15-20% with emergency colectomy in the setting of gangrenous bowel or sepsis, compared with significantly lower mortality for elective procedures on viable bowel, underscoring the importance of early recognition and timely decompression to avoid catastrophic progression and minimize colonic ischemia [17]. In the pediatric literature, early endoscopic decompression followed by definitive resection during the index hospitalization has been associated with favorable outcomes and low recurrence, although the small case numbers limit robust comparative analyses of specific operative techniques [10,15]. Intraoperative confirmation of a redundant sigmoid colon with a narrow mesenteric attachment and absence of chronic inflammatory or motility disorders further supports the hypothesis that primary anatomic variation alone can predispose otherwise healthy adolescents to SV, and that definitive resection reduces the risk of recurrence [2,5,6,15-19].

## Conclusions

Pediatric SV is a rare but emergent cause of large bowel obstruction that may present with highly atypical symptoms, including isolated bilateral low back and flank pain with only subtle or transient abdominal complaints. In adolescents with new-onset low back pain and bowel incontinence, clinicians should maintain a high index of suspicion for intra-abdominal pathology and avoid anchoring prematurely on benign musculoskeletal diagnoses, as delays in recognition can allow a reversible volvulus to progress to catastrophic ischemic perforation. When SV is suspected, urgent surgical or gastroenterology consultation with staged management, including endoscopic decompression followed by definitive sigmoid resection during the same admission, can optimize outcomes and minimize the risk of recurrence.
